# Satellite sensor requirements for monitoring essential biodiversity variables of coastal ecosystems

**DOI:** 10.1002/eap.1682

**Published:** 2018-03-06

**Authors:** Frank E. Muller‐Karger, Erin Hestir, Christiana Ade, Kevin Turpie, Dar A. Roberts, David Siegel, Robert J. Miller, David Humm, Noam Izenberg, Mary Keller, Frank Morgan, Robert Frouin, Arnold G. Dekker, Royal Gardner, James Goodman, Blake Schaeffer, Bryan A. Franz, Nima Pahlevan, Antonio G. Mannino, Javier A. Concha, Steven G. Ackleson, Kyle C. Cavanaugh, Anastasia Romanou, Maria Tzortziou, Emmanuel S. Boss, Ryan Pavlick, Anthony Freeman, Cecile S. Rousseaux, John Dunne, Matthew C. Long, Eduardo Klein, Galen A. McKinley, Joachim Goes, Ricardo Letelier, Maria Kavanaugh, Mitchell Roffer, Astrid Bracher, Kevin R. Arrigo, Heidi Dierssen, Xiaodong Zhang, Frank W. Davis, Ben Best, Robert Guralnick, John Moisan, Heidi M. Sosik, Raphael Kudela, Colleen B. Mouw, Andrew H. Barnard, Sherry Palacios, Collin Roesler, Evangelia G. Drakou, Ward Appeltans, Walter Jetz

**Affiliations:** ^1^ College of Marine Science University of South Florida 140 7th Avenue South Saint Petersburg Florida 33701 USA; ^2^ School of Engineering University of California Merced 5200 N. Lake Road Merced California 95340 USA; ^3^ Joint Center for Earth Systems Technology University of Maryland 5523 Research Park Drive Baltimore Maryland 21228 USA; ^4^ Department of Geography University of Southern California Santa Barbara California 93106 USA; ^5^ Applied Physics Lab Johns Hopkins University 11100 Johns Hopkins Road Laurel Maryland 20723 USA; ^6^ Scripps Institution of Oceanography University of California San Diego La Jolla California 92093 USA; ^7^ Commonwealth Scientific and Industrial Research Organisation Canberra Australian Capital Territory Australia; ^8^ Stetson University College of Law 1401 61st Street South Gulfport Florida 33707 USA; ^9^ HySpeed Computing Miami Florida 33143 USA; ^10^ U.S. Environmental Protection Agency National Exposure Research Laboratory Research Triangle Park Raleigh North Carolina 27711 USA; ^11^ Ocean Ecology Laboratory Goddard Space Flight Center National Aeronautics and Space Administration Greenbelt Maryland 20770 USA; ^12^ Goddard Space Flight Center Science Systems and Applications Greenbelt Maryland 20770 USA; ^13^ Naval Research Laboratory Washington D.C. 20375 USA; ^14^ Department of Geography University of California Los Angeles Los Angeles California 90095 USA; ^15^ Goddard Institute for Space Studies Columbia University New York New York 10025 USA; ^16^ City University of New York New York New York 10031 USA; ^17^ School of Marine Sciences University of Maine Orono Maine 04469 USA; ^18^ Jet Propulsion Laboratory California Institute of Technology Pasadena California 91109 USA; ^19^ Universities Space Research Association Goddard Space Flight Center National Aeronautics and Space Administration Greenbelt Maryland 20770 USA; ^20^ NOAA Geophysical Fluid Dynamics Laboratory Princeton New Jersey 08540 USA; ^21^ Climate and Global Dynamics Laboratory University Corporation for Atmospheric Research Boulder Colorado 80301 USA; ^22^ Laboratorio de Sensores Remotos Universidad Simon Bolívar Sartenejas, Apartado Caracas 89000 Venezuela; ^23^ Lamont Doherty Earth Observatory Columbia University Palisades New York 10964 USA; ^24^ College of Oceanic and Atmospheric Science Oregon State University Corvallis Oregon 97331 USA; ^25^ Roffer's Ocean Fishing Forecasting Service 60 Westover Drive, West Melbourne Florida 32904 USA; ^26^ Alfred Wegener Institute Helmholtz Centre for Polar and Marine Research Bremerhaven Germany; ^27^ Stanford University Stanford California 94305 USA; ^28^ Department of Marine Sciences University of Connecticut Groton Connecticut 06340 USA; ^29^ Earth System Science and Policy University of North Dakota Grand Forks North Dakota 58202 USA; ^30^ Bren School of Environmental Science and Management University of California Santa Barbara California 93106 USA; ^31^ EcoQuants 508 East Haley Street Santa Barbara California 93103 USA; ^32^ Florida Museum of Natural History University of Florida Cultural Plaza, 3215 Hull Road Gainesville Florida 32611 USA; ^33^ Wallops Flight Facility NASA Goddard Space Flight Center Wallops Island, Virginia 23337 USA; ^34^ Woods Hole Oceanographic Institution Woods Hole Massachusetts 02543 USA; ^35^ University of California Santa Cruz Santa Cruz California 95064 USA; ^36^ Graduate School of Oceanography University of Rhode Island Kingston Rhode Island 02881 USA; ^37^ WET Labs/Sea‐Bird Scientific P.O. Box 518 Philomath Oregon 97370 USA; ^38^ Airborne Science Program NASA Ames Research Center Moffett Field California 94035 USA; ^39^ Department of Earth and Oceanographic Science Bowdoin College Brunswick Maine 04011 USA; ^40^ Geo‐Information Science and Earth Observation (ITC) University of Twente Enschede The Netherlands; ^41^ Intergovernmental Oceanographic Commission of UNESCO Ocean Biogeographic Information System Oostende Belgium; ^42^ Department of Ecology and Evolutionary Biology Yale University New Haven Connecticut 06511 USA

**Keywords:** aquatic, coastal zone, ecology, essential biodiversity variables, *H4* imaging, hyperspectral, remote sensing, vegetation, wetland

## Abstract

The biodiversity and high productivity of coastal terrestrial and aquatic habitats are the foundation for important benefits to human societies around the world. These globally distributed habitats need frequent and broad systematic assessments, but field surveys only cover a small fraction of these areas. Satellite‐based sensors can repeatedly record the visible and near‐infrared reflectance spectra that contain the absorption, scattering, and fluorescence signatures of functional phytoplankton groups, colored dissolved matter, and particulate matter near the surface ocean, and of biologically structured habitats (floating and emergent vegetation, benthic habitats like coral, seagrass, and algae). These measures can be incorporated into Essential Biodiversity Variables (EBVs), including the distribution, abundance, and traits of groups of species populations, and used to evaluate habitat fragmentation. However, current and planned satellites are not designed to observe the EBVs that change rapidly with extreme tides, salinity, temperatures, storms, pollution, or physical habitat destruction over scales relevant to human activity. Making these observations requires a new generation of satellite sensors able to sample with these combined characteristics: (1) *spatial resolution* on the order of 30 to 100‐m pixels or smaller; (2) *spectral resolution* on the order of 5 nm in the visible and 10 nm in the short‐wave infrared spectrum (or at least two or more bands at 1,030, 1,240, 1,630, 2,125, and/or 2,260 nm) for atmospheric correction and aquatic and vegetation assessments; (3) *radiometric quality* with signal to noise ratios (SNR) above 800 (relative to signal levels typical of the open ocean), 14‐bit digitization, absolute radiometric calibration <2%, relative calibration of 0.2%, polarization sensitivity <1%, high radiometric stability and linearity, and operations designed to minimize sunglint; and (4) *temporal resolution* of hours to days. We refer to these combined specifications as *H4* imaging. Enabling *H4* imaging is vital for the conservation and management of global biodiversity and ecosystem services, including food provisioning and water security. An agile satellite in a 3‐d repeat low‐Earth orbit could sample 30‐km swath images of several hundred coastal habitats daily. Nine *H4* satellites would provide weekly coverage of global coastal zones. Such satellite constellations are now feasible and are used in various applications.

## Introduction

Water and life: no two features more completely define planet Earth and no two are more inextricably intertwined. This link is especially strong in the coastal zone, where life is diverse and productive at many levels of the food web. The physical and biological elements of coastal habitats can change rapidly with many types of disturbance, such as extreme tides, extreme temperatures, extreme high or low salinities, severe storms, and human use including pollution or physical destruction. Yet monitoring changes in coastal habitats has been difficult. Field measurements on land or in adjacent shallow aquatic areas can be detailed and of high quality, but they are often limited by temporal frequency. Additionally, because they are expensive and hard to conduct, these studies and surveys typically cover only small areas. Thus, for the most part, the highly variable aquatic and emergent elements of coastal habitats, including wetlands, remain among the most undersampled habitats on the Earth's surface. Many terrestrial ecosystems, including freshwater ones, are just as diverse and difficult to monitor as coastal aquatic areas. They contain mosaics of different habitats with assorted substrates and living elements spread over scales spanning tens of meters to kilometers. They can change rapidly due to the overlap in phenologies of different populations of organisms, or because of a disturbance such as fires or hurricanes.

Characterizing these habitats in a manner that is relevant to scientific, conservation, and other socioeconomic goals requires measurements that are sensitive to temporal changes, cost effective, and allow for an assessment across large spatial scales. These criteria are the basis for Essential Climate Variables (Bojinski et al. 2014) and for systematic ecological observations using Essential Biodiversity Variables (EBV; Pereira et al. 2013). To characterize the diversity, composition, and function of both terrestrial and coastal aquatic ecosystems, these observations need to be acquired synoptically. We outline specifications for satellite remote sensing of coastal measurements that offer the potential for rapid, frequently repeated, and consistent high‐quality observations to characterize changes in EBVs across a wide range of terrestrial and aquatic ecosystems. We specifically address EBVs relevant to community composition and trait diversity. We refer to this remote sensing strategy as *H4* imaging because it is based on the combined requirements for high spatial, temporal, and spectral resolution, as well as high radiometric quality.

## The Relevance of the Coastal Zone

Humanity benefits directly from marine resources concentrated along the coast, including economic value, clean water, food, energy, pharmaceuticals, and space for recreation (Hay and Fenical 1996, Mimouni et al. 2012, Malve 2016). Areas within 100 km of the coast provide benefits equivalent to over 60% of the world's total gross national product, or over US$26 trillion every year (MEA 2005a). These coastal areas include diverse wetland ecosystems, which are broadly defined as biologically structured habitats where water saturation is a dominant factor in determining the plant and animal communities that occupy these areas. The definition for wetlands used by the Ramsar Convention includes rocky shores, coral reefs, and sea grasses to a depth of 6 m at low tide (Scott and Jones 1995, Finlayson 2016). This definition is loosely based on the classification developed by Cowardin et al. (1979) for the U.S. government. Coastal wetlands alone provide over US$15 trillion in annual benefits, including significant protection to human life and property (MEA 2005b, Barbier 2016, Narayan et al. 2017). Yet, between 30% and 70% of wetlands were lost in the 20th century as a result of development, pollution, poor water management, and overfishing (Bruland 2008, Bromberg‐Gedan et al. 2009, Davidson 2014, Hu et al. 2017). An additional 20–70% of coastal wetlands could be lost by 2080 because of sea level rise and continuing human‐related pressures (Nicholls 2004, Gardner et al. 2015).

Many of the benefits that we derive from coastal ecosystems depend on the number of species, the abundance and biomass of organisms, the diverse interactions between organisms and the environment, and the number of different habitats in these areas (Malone et al. 2013). We have increasing evidence that biomass production increases with species richness in a wide range of marine and terrestrial ecosystems and not simply in response to abiotic effects (Duffy et al. 2017). Moreover, changes in the community composition of lower trophic levels can have major impacts on higher trophic levels, determining the success or loss of animal populations such as fish, waterfowl, and marine mammals (Platt et al. 2003, Ji et al. 2010, Wood and Kellermann 2015, Santora et al. 2017). Top‐down pressures due to the harvesting of top predators and other higher trophic levels also often have impacts that can cascade down the food web (Pace et al. 1999). Changes in climatic factors such as temperature and rainfall, and human activity, can also affect species ranges and promote invasive species in aquatic bodies and on land (Andrew and Ustin, 2008).

**Figure 1:**
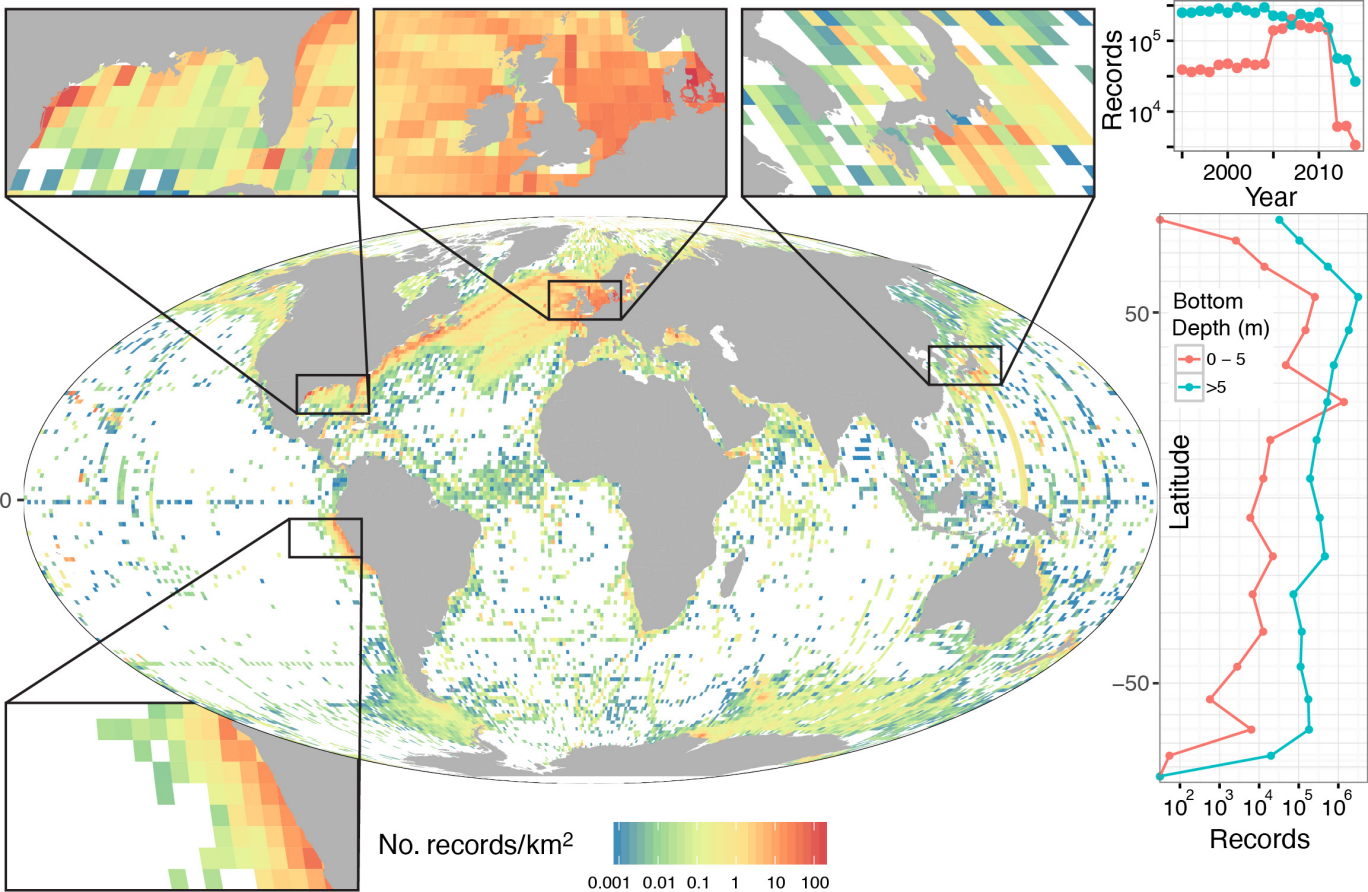
The Ocean Biogeographic Information System (OBIS 2017) is the preeminent open‐access database for international marine biodiversity assessments. This map shows the density of taxonomic records from the OBIS in 1 × 1° cells of the global ocean in near‐surface pelagic and coastal waters (upper 20 m; *n* = 10.8 million; Mollweide projection map of the number of records per square kilometer; color bar in log_10_‐scale; data extracted 3 October 2016). Nearshore records represent benthic and water column data combined in waters from 0 m to 5 m bottom depth. Pelagic records are sampled from the surface ocean (upper 20 m) starting at a bottom depth of 5 m near the coast. The four inset maps show regions around the globe with dense OBIS records, yet these also demonstrate inconsistent spatial coverage. Right‐hand graphics: The shallow pelagic records (>5 m bottom depth) generally show two to three orders of magnitude more observations than nearshore areas in most latitude bands. The sudden increase in nearshore records in the 2005–2010 timeframe is largely a contribution of observations collected in the Florida Keys region (USA). The overall decline in data after 2010 highlights typical delays in processing and reporting biological observations to OBIS. Systematic sampling by satellite remote sensing, combined with field observations, animal tracking, and modeling, promise to fill the widespread gaps in space and time and enable routine assessments of marine biodiversity in the world's coastal and pelagic zones.

Characterizing how community structure and the phenology of organisms that use coastal ecosystems shifts due to human activities, biotic interactions, and processes associated with a changing climate is a core focus of current scientific research. Indeed, among the highest priority research questions in coastal ecology are: How will the diversity of life in coastal zones change with climate and with increased human uses? How will these changes affect the ecology and biogeochemistry of coastal and other marine habitats? What are the relationships between species diversity and ecosystem function? Addressing these questions is key to tracking progress toward conservation, management, and sustainable development (e.g., United Nations 2015; Agenda 2015). However, today it is difficult to address these questions because measurements of biodiversity are often limited in temporal frequency and typically cover only small areas. Many coastal habitats are also remote or difficult to access, further limiting sampling opportunities. For example, the Ocean Biogeographic Information System (OBIS; Appeltans et al. 2012, see OBIS 2017), the preeminent open‐access database for international marine biodiversity observations, shows large areas of the coast and the surface ocean with no data (Fig. 1). Information latency is also slow: there is a 5–10 year lag before research data are delivered to OBIS (Fig. 1, inset). This seriously hampers the ability to monitor for change and any possible national or international response to an environmental issue.

**Figure 2:**
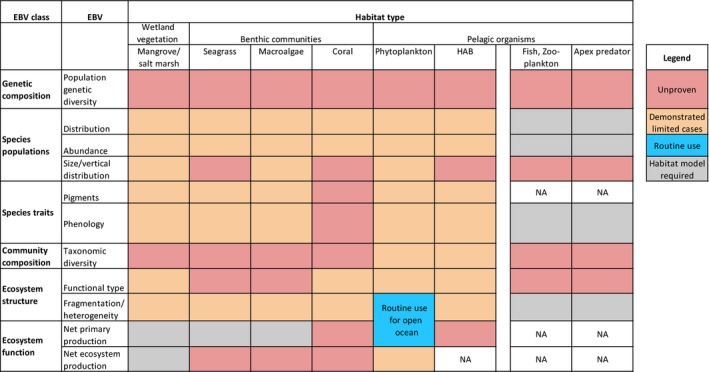
Current capabilities of remotely sensed data for measuring Essential Biodiversity Variables (EBVs; Pereira et al. 2013). The EBVs are complementary to the GOOS Essential Ocean Variables for biology and ecology (FOO 2012). “Unproven” indicates that methods have not yet been developed to collect these measurements from satellite/aerial data. “Demonstrated in limited cases” are methods that have been demonstrated and that could be made operational with the proposed *H4* imaging approach. “Routine use” indicates measurements that are produced regularly, and at present include distribution, abundance, and phenology of bulk phytoplankton only in the open ocean (i.e., derived chlorophyll *a* concentration). “Habitat model required” indicates EBVs that can be predicted on the basis of habitat correlations developed from remotely sensed data. “NA” indicates that the observation is not applicable.

Answering the fundamental ecology questions previously mentioned requires characterizing and detecting change in specific elements of coastal ecosystems, including factors that can be the environmental and human drivers of change. For example, monitoring the diversity of life and detecting change in the ecology and biogeochemistry of coastal zones requires monitoring EBVs, such as groups of species populations, traits of assemblages of species, and community properties (Fig. 2). Understanding and explaining ecological change requires the context of long‐term measurements of environmental parameters such as temperature, discharge, and indicators of water quality, as well as quantifying anomalies in these parameters. Monitoring ecosystem structure EBVs (Fig. 2) also requires assessing changes in human activities, as these factors may lead to ecosystem change. Furthermore, EBVs have to be estimated consistently over large areas and all around the world, which is only possible by complementing in situ measurements with those collected from the vantage point of Earth‐observing satellites.

## Characteristic Scales of Variation in Coastal Zones

**Figure 3:**
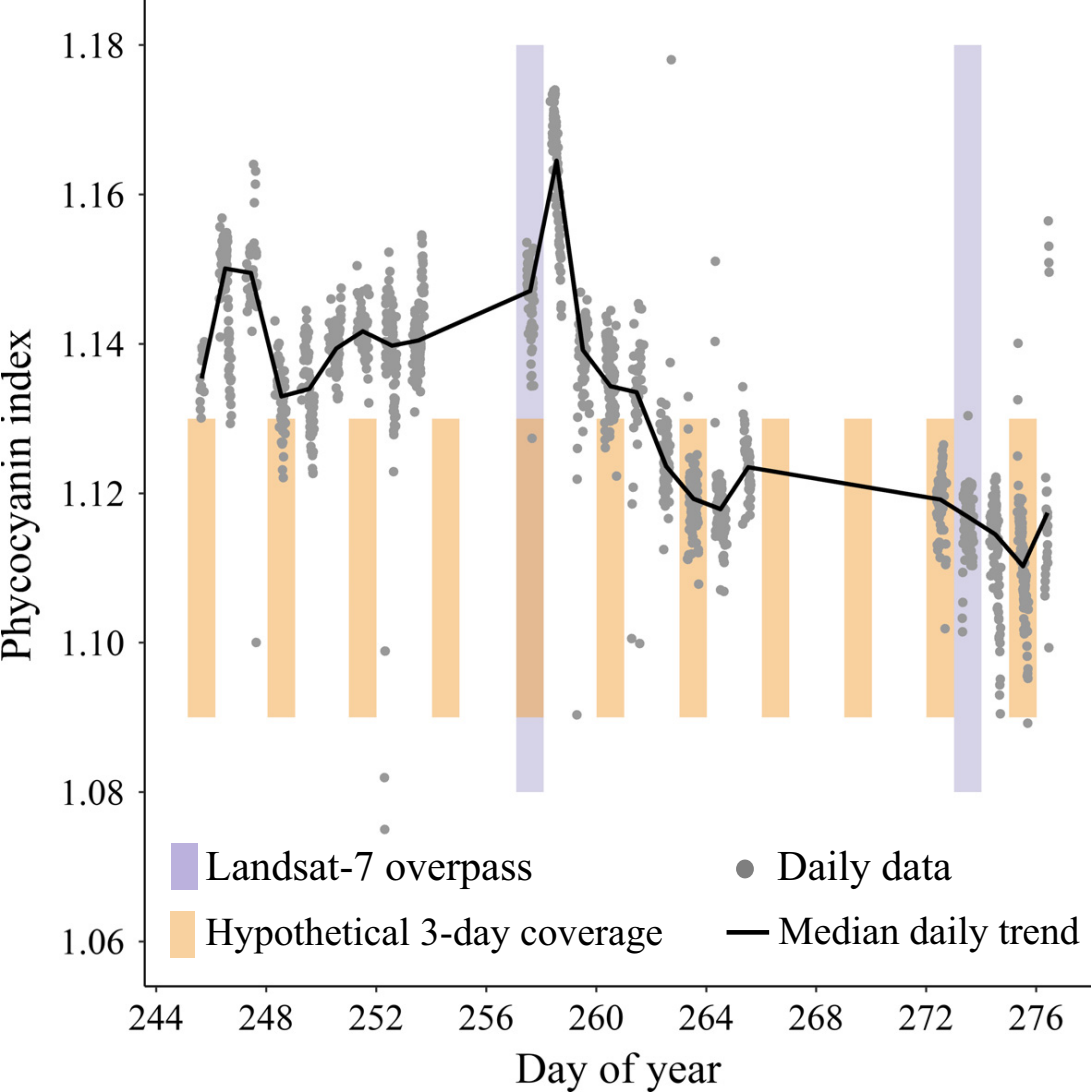
Illustration of rapid changes in concentration of nuisance cyanobacteria, quantified as a phycocyanin pigment index. In situ measurements conducted every 15 minutes on a daily basis with a hand‐held spectrometer were used to identify the organism in Upper Mantua Lake (Italy). Gaps in the time series are due to night and cloudy days. The frequency of sampling of a Landsat sensor (16 d), shown as gray vertical bars, would alias changes in the concentration of phytoplankton, sediment load, and other water quality factors. Orange vertical bars illustrate a 3‐d sample frequency, i.e., five times the Landsat frequency. Some species of cyanobacteria can outcompete other phytoplankton, form noxious or toxic blooms, and ultimately reduce water quality for the rest of the food web and human consumption (after Hestir et al. 2015).

Phytoplankton communities and their concentrations in coastal and inland waters often change over scales of hours to days due to runoff, advection, mixing due to tides, currents, and winds, and to biotic interactions (Chen et al. 2010, Tzortziou et al. 2011, Moreno Madriñán et al. 2012). Several case studies have used spectrometers and other bio‐optical devices deployed on platforms such as towers, boats, and aircraft to measure rapid changes in biodiversity and phenology in such conditions (Pengra et al. 2007, Adam et al. 2010, Lantz 2012). For example, Hestir et al. (2015) documented changes in the concentration of cyanobacteria in lakes in Italy over scales of days with field spectroscopy data (Fig. 3). Kudela et al. (2015) used field spectroscopy observations to show that phytoplankton blooms can be displaced by toxic cyanobacteria in only a few days in Pinto Lake, California. In order to detect long‐term trends, such measurements of short‐term variability are required over long periods of time. An excellent example of trends in an aquatic ecosystem was provided by Hunter‐Cevera et al. (2016). They detected shifts in the timing of annual blooms of the phytoplankter *Synechococcus* with an automated submersible flow cytometer deployed at the Martha's Vineyard Coastal Observatory. Spring blooms occurred progressively earlier in the season as temperatures became warmer, and by 2012, the blooms began up to 20 d earlier than they had in 2003. At higher latitudes, shifts toward phytoplankton species more typical of warmer waters have also been documented (Hays et al. 2005, Dybas 2006).

Field studies of Nordic wetlands spectra show significant changes in vegetation colors in less than a week (Eklundh et al. 2011). Indeed, wetland species, including invasive species, can be identified by the change of spectral signatures over the growing cycle (Gilmore et al. 2008, Ouyang et al. 2013). These observations also demonstrate that phenology is a sensitive indicator of environmental change, but that observing such changes in phytoplankton or wetland vegetation requires sampling at frequencies on the order of a week or faster to differentiate seasonal or longer‐term changes relative to short‐term variability.

The spatial variability of coastal habitats is also high. Dominant spatial variability of physical, biological, geological, and biogeochemical properties of coastal waters changes with distance from the coast (Bissett et al. 2004). In terms of horizontal distribution, close to the coast, these properties tend to have peak variability at between 70 and 600 m. Farther offshore, out to about 5 km off the coast, features such as fronts and phytoplankton blooms show high variability around 100–200 m. Observing and monitoring these features and their variability requires sampling at between about 30 and 100 m (Moses et al. 2016). At distances larger than 10 km from the coast, features show typical scales of 1 km or larger, which can be detected with coarser resolution sensors (Bissett et al. 2004). Wetland habitats show variability at smaller spatial scales. Turpie et al. (2015) studied the impact of varying spatial resolution on mapping of coastal tidal wetland habitats. They concluded that a spatial resolution of approximately 30‐m pixels or smaller is ideal to map wetlands. Coarser spatial resolution sensors smear and confound spectral and spatial patterns necessary to identify biota and quantify habitat variability.

These spatial scales are sampled adequately by current sensors such as the Operational Land Imager (OLI) on the Landsat 8 satellite, operated by the U.S. Geological Survey, and the MultiSpectral Instrument (MSI) on Sentinel 2A/B, operated by the European Space Agency under the Copernicus program (Vanhellemont and Ruddick 2015, Pahlevan et al. 2017a). The combination of Landsat 8/OLI and Sentinel 2A/B allows the development of applications that require relatively high temporal frequency, i.e., observations every 4 d or more frequent. However, this sensor class lacks the spectral definition in the visible and near‐infrared light (i.e., spectral resolution of 5 nm or better between 380 nm and 900 nm, and about 10 to 20 nm between 900 and 2500 nm) needed to estimate the biodiversity of coastal organisms and habitats. Other satellite sensors meet the required 5–10 nm spectral resolution, but lack in spatial detail, such as the 1‐km spatial resolution planned for the PACE ocean color sensor (PACE SDT 2012).

The NASA Hyperspectral Infrared Imager (HyspIRI) mission concept, the JAXA HISUI instrument, and the DLR Environmental Mapping and Analysis Program (EnMAP; Guanter et al. 2015) will also have 30‐m spatial resolution (Turpie et al. 2015). HyspIRI is being designed to sample nominally every 16 d, and EnMAP and HISUI are designed to acquire targets of interest intermittently. Thus, they will lack temporal detail needed to observe changes over the scale of days.

The studies just described show that aspects of biodiversity and phenology are observable with remote sensing globally and across a range of time and spatial scales using bio‐optical methods. A recent extensive feasibility study conducted on behalf of the Committee on Earth Observing Satellites (CEOS 2017) concluded that imaging spectrometers are the desired tool to conduct terrestrial and ocean remote sensing of freshwater, estuarine, and coastal environments to characterize water quality, bathymetry, and benthic habitats.

## Essential Biodiversity Variables in the Coastal Zone

Pereira et al. (2013; see also Geijzendorffer et al. 2015, Pettorelli et al. 2016, Kissling et al. 2017) proposed that EBVs can be grouped into six classes: genetic composition, species populations, species traits, community composition, ecosystem structure, and ecosystem function. Fig. 2 highlights the classes of EBVs that are well suited for remote sensing applications, such as species populations, species traits, and ecosystem structure. There is a potential to expand the number of EBVs that can be measured today by measuring surface spectral reflectance of visible and near‐infrared light (i.e., from 380 nm to 2500 nm). The EBVs are complementary to the Essential Ocean Variables (EOVs) defined by the Global Ocean Observing System (GOOS) in its Framework for Ocean Observing (FOO, 2012). The spectral reflectance contains the signatures of specific traits of groups of species populations or habitat elements, defined by their absorption, scattering, and fluorescence emission characteristics (Colgan et al. 2012, Asner et al. 2017. Kissling et al. (2017) emphasized that progress in defining these EBVs is stimulated by the coordinated collection and sharing of in situ biodiversity observations (e.g., Jetz et al. 2012) and open access to satellite data sets (e.g., Skidmore et al. 2015). Indeed, in situ data are fundamental to algorithm development efforts that link observable geophysical quantities and EBVs.

Satellite sensors can cover large areas quickly and repeatedly. Estimates of wetland extent have been periodically generated from Landsat since the early 1970s (Tiner et al. 2015, McCombs et al. 2016). In this time frame, satellite instruments have also routinely measured ocean currents, surface winds, precipitation, and color and temperature of the ocean surface (Muller‐Karger et al. 2013). These observations have resulted in an unprecedented understanding of physical changes in the environment and have advanced our knowledge of coastal and oceanic ecosystems. State of the art remote sensing research focused on marine biodiversity includes open ocean detection of diatoms and their phenology (Racault et al. 2012, IOCCG 2014, Soppa et al. 2016), tracking of harmful algal blooms (e.g., Soto et al. 2016), and testing of algorithms for phytoplankton size distribution and functional group detection (Uitz et al. 2010, Mouw et al. 2012, Brotas et al. 2013, Bracher et al. 2017). Remote sensing is also critically important to map and monitor coral reef extent and health (Andréfouët et al. 2005), but there remain fundamental problems in the discrimination between coral and benthic algae (Hedley et al. 2016a).

Governments around the world, organized under the Group on Earth Observations Biodiversity Observation Network (GEO BON), are defining strategies to estimate EBVs from space. However, we cannot obtain key information to evaluate the EBVs of coastal aquatic and wetland habitats shown in Fig. 2 from current or past satellite sensors. These sensors have shortcomings in their combined spectral, spatial, and/or temporal resolution (Hestir et al. 2015, Bracher et al. 2017, CEOS 2017).

Remote sensing is an important tool to monitor anthropogenic activities (e.g., land use and cover change, oil spills) and their impact in coastal zones (Muller‐Karger et al. 2014, CEOS 2017). Remote sensing also offers significant potential to help in the design and management of marine protected areas (Kachelriess et al. 2014). These applications require measuring the condition of marine habitats, including water quality, sea surface temperature, currents, and eddies, and assessing the spatial extent of biologically structured habitats (reefs, seagrass meadows, mangrove forests, salt marshes, etc.). These factors can all affect species diversity and productivity of these systems. Since the launch of the Coastal Zone Color Scanner (CZCS; Gordon and Morel 1983) and the first Landsat sensors (Tiner et al. 2015) in the 1970s, the coastal zone has been observed remotely with multispectral imaging missions designed either for bright terrestrial targets or relatively dark targets such as the surface of the open ocean. Sensors launched since then lack either the spectral, temporal, or the spatial resolution to observe ecological characteristics of coastal habitats, and therefore cannot be used to identify assemblages of species populations, measure the fast changes of communities living in coastal areas, or evaluate the spatial structure and integrity of typical coastal aquatic and wetland habitats. No space‐based mission has yet been designed to study and monitor the canopy to benthos continuum of global coastal habitats (Dekker and Pinnel 2017).

## Essential Biodiversity Variables in Open Ocean Habitats

We currently derive bulk phytoplankton pigment and carbon concentration in the pelagic global ocean from satellite ocean color measurements with a spatial resolution of about 1 km (Fig. 2). Since 1996, these estimates have been made using observations collected from a series of sensors. Long term (i.e., decade‐long) records of ocean color are crucial to assess the effects of natural and anthropogenic changes on oceans. The National Oceanic and Atmospheric Administration (NOAA) plans to continue the Visible Infrared Imaging Radiometer Suite (VIIRS) series on future Joint Polar Satellite System (JPSS) platforms, but this sensor does not measure radiance in the red absorption wavelengths of chlorophyll, in wavelengths of absorption by phycobiliproteins characteristic of cyanobacteria, or the solar‐stimulated fluorescence of phytoplankton. This limits the ability to identify phytoplankton blooms in coastal waters affected by river discharge, where colored dissolved organic matter (CDOM) masks the blue absorption features in the spectral signature of chlorophyll. The U.S. National Aeronautics and Space Administration (NASA) Plankton, Aerosol, Cloud, and ocean Ecosystem (PACE) mission will cover key gaps in the visible color spectrum (PACE SDT 2012). PACE will have a nominal spatial resolution of 1 km and a spectral resolution of 5 nm from the ultraviolet to the near infrared. This could improve our ability to monitor biodiversity in pelagic ocean waters by quantifying phytoplankton functional types (IOCCG 2014), including nitrogen‐fixing organisms (e.g., *Trichodesmium*), calcifiers (coccolithophores), producers of dimethyl sulfide or DMS (e.g., *Phaeocystis*), silicifiers (e.g., diatoms), and harmful algal blooms.

PACE is expected to launch in the 2022–2023 timeframe and conduct observations over 3 to 10 years. In addition, the European Space Agency has launched Sentinel‐3A, and will soon launch Sentinel‐3B in 2018, containing the two multispectral Ocean and Land Colour Instruments (OLCI; 22 spectral bands each). These are part of the Copernicus program, and together enable global ocean coverage every 1.5 d at 300‐m spatial resolution, not accounting for clouds. While the Sentinel‐3 A/B OLCI and PACE sensors offer improved capabilities to observe the global ocean, they are not designed to monitor coastal ecosystems. In coastal areas, the influence of the seafloor, land areas, and constituents that affect water quality are often confounded in the signals recorded by these coarse spatial resolution imaging devices. Thus, despite the advances and benefits provided by these instruments, another class of sensors is required to adequately observe coastal zones.

## Requirements for Observing Coastal Biodiversity and Ecosystem Change

Directly measuring EBVs (Fig. 2) across the coastal zones of the world requires repeated observations of areas spanning hundreds to thousands of square kilometers at a spatial resolution adequate to detect change across environmental gradients in aquatic and adjacent wetland settings. This requires sampling with stringent specifications in four categories: *spatial resolution*,* spectral resolution*,* radiometric quality*, and *temporal resolution*. We refer to this demanding strategy as *H4* sensing. We examine each of these required dimensions below.

### High spatial resolution

As mentioned above, Turpie et al. (2015) concluded that a spatial resolution higher than 30‐m pixels is ideal to observe the emergent vegetation of coastal wetlands. This is an adequate resolution to map submerged biologically structured habitats like coral reefs and sea grass beds (Andréfouët et al. 2005, Wabnitz et al. 2010, Hedley et al. 2016a). To characterize coastal phytoplankton blooms, surface floating vegetation, suspended particulate matter, and colored dissolved matter, about 100‐m or smaller pixels are needed (Bissett et al. 2004, Dierssen et al. 2015a, Hedley et al. 2016b, Moses et al. 2016). The CEOS (2017) report considers that a global mapping mission for aquatic ecosystem biogeochemistry, including coastal marine and freshwater bodies such as rivers requires a spatial resolution significantly higher than 250‐m. However, some applications, for example monitoring coral bleaching events, require a much higher spatial resolution (Andréfouët et al. 2002; CEOS 2017).

### High spectral resolution

High spectral resolution has several benefits. NASA's Hyperion sensor operated on the Earth Observing‐1 (EO‐1) satellite as a technology demonstration between 2000 and 2017. It provided 30‐m spatial resolution images with 220 bands from 400 to 2,500 nm at 10‐nm resolution and with signal‐to‐noise ratios intended for imaging bright land targets. Hyperion demonstrated the potential of high spectral resolution data to derive bathymetry, identify bottom types, and discriminate between wetland species in different coastal areas (Brando and Dekker 2003, Pengra et al. 2007). Pahlevan and Schott (2013) also demonstrated the higher‐quality of Hyperion‐derived chlorophyll *a* concentrations compared to those derived from simulated Landsat sensors near the Niagara River discharge. In 2009, the U.S. Office of Naval Research and NASA installed the Hyperspectral Imager for the Coastal Ocean (HICO) on the International Space Station (ISS; Davis and Tufillaro 2013). HICO had a spectral resolution of 5.7 nm from 400 to 900 nm, a spatial resolution of 100 m, and a very infrequent revisit time for observing the same target on the ground. These acquisition limitations were in part due to the low‐inclination orbit of the ISS, periodic maneuvers to raise and lower the space station, and other operational scheduling concerns. Although HICO ceased operations in 2014, it demonstrated the potential of high spectral resolution to derive bathymetry, bottom types, water optical properties, phytoplankton bloom types, suspended sediment type, and wetland vegetation maps (Ryan et al. 2014). High spectral resolution also enables algorithm development and the synthetic spectral reconstruction of different satellite sensor bands (e.g., Osterman et al. 2016).

High spectral resolution is also required to separate aquatic constituents by their light absorption, scattering, and fluorescence characteristics (PACE SDT 2012). These include chlorophyll *a* absorption at 435–438 nm, other accessory pigment absorption features between 550 and 900 nm, and fluorescence by chlorophyll *a* and other pigments (Dierssen et al. 2015b, Hu et al. 2005, Chase et al. 2017). A minimum spectral resolution of 6–8 nm is required in the visible wavelengths to separate diagnostic accessory pigments of phytoplankton as well as fluorescence signals in the reflectance spectrum (Dekker and Pinnel 2017). Other derived products include CDOM and sediment concentration. Higher spectral resolution also allows more spectral benthic cover types to be discriminated to deeper depths (Botha et al. 2013). Additional EBVs of interest that may be derived from high spatial and spectral resolution data are coral, macrophyte, and wetland extent (Fig. 2).

Deriving EBVs for coastal habitats therefore requires measurements at ~5 nm resolution in the visible (VIS; 340–900 nm spectral range) and at ~10 nm resolution in the short‐wave infrared (SWIR; 900–2500 nm; or at least two or more bands at 1,030, 1,240, 1,630, 2,125, and 2,260 nm). The SWIR measurements are required for differentiating wetland vegetation communities (Vaiphasa et al. 2005, Hestir et al. 2012) and are particularly critical for atmospheric correction algorithms over turbid waters (Jiang and Wang 2014, Frouin and Pelletier 2015, Pahlevan et al. 2017b). To that end, atmospheric correction approaches for a coastal mission can leverage the maturity of operational algorithms for ocean color missions (Ahmad et al. 2010), but need to be updated to address coastal and inland aerosol types (Pahlevan et al. 2017b), hyperspectral data, and higher spatial resolution. Atmospheric correction should also incorporate procedures to evaluate and correct sun glint (e.g., Steinmetz et al. 2011, Devred et al. 2013, Botha et al. 2016) and the radiance reflected from adjacent pixels (adjacency effect; e.g., Duan et al. 2015).

### High radiometric quality

Retrieving estimates of constituent concentrations with better than 20% accuracy requires signal‐to‐noise ratios similar to those proposed for PACE (Hu et al. 2012). Specifically, the NASA PACE Science Definition Team (PACE SDT 2012) concluded that ocean observations require a sensor with signal‐to‐noise ratios (SNR) >1000 for visible radiance bands for signal levels typically observed over open ocean waters, absolute radiometric calibration ≪2%, and relative calibration of 0.2%. These requirements are more critical at higher latitudes due to lower sun angles (Dekker and Pinnel 2017). In contrast, the existing high spatial resolution missions, including Landsat 8 and Sentinel 2A/B, have SNRs on the order 300–400 in the 443‐nm channel and lower in the longer wavelengths (Pahlevan et al. 2014, 2017a,2017b). The SNR of such sensors can be improved by aggregating pixels and degrading spatial resolution. As of 2018, PACE‐like SNR for aquatic biogeochemistry observations may be achievable at 100‐m or finer spatial resolution.

Different coastal waters exhibit low radiance values in different parts of the spectrum and these values change with time due to the co‐occurrence of different colored submerged vegetation, phytoplankton, other particulate and dissolved substances, and bottom depth. Because of the very high dynamic range of reflected radiances across the spectrum from different coastal aquatic habitats, there is no typical radiance to use as a standard to define a SNR specification. This wide range of radiances reflected by coastal habitats, from very dark to very bright, requires the highest sensitivity possible. We therefore recommend SNR above 800 based on signal levels typical of the open ocean.

Other radiometric considerations include the following: 14‐bit digitization, absolute radiometric calibration <2%, and relative calibration 0.2% with sensor radiometric stability and linearity, and strategies to monitor these characteristics. All spectral bands of a scene should be registered simultaneously. Further, aquatic observations require minimal polarization sensitivity (<1%), with carefully characterized polarization response. Stray light, spectral out‐of‐band, and crosstalk signals, including instrument response‐versus‐scan, spectral smile (spectral distortion or shift along a sensor scan line), and residual polarization should be minimal, and should be carefully monitored over time. In general, on‐orbit variation in instrument radiometric response with time should be monitored and adjusted. Sustained calibration needs to include frequent observations of the Moon (e.g., once per day over at least half of the lunar cycle), stable on‐board reference standards, and vicarious calibration and product validation efforts. Observations must include an active sun glint avoidance and mitigation strategy, such as tilting <20° from surface specular reflection. The platform should also exhibit minimal jitter with high pointing accuracy, and accurate band‐to‐band registration. Furthermore, standard and reference in situ radiometric measurements such as those available from the Marine Optical BuoY (MOBY; Clark et al. 2003), should be available for mission‐long vicarious calibration.

### High temporal resolution

Observations at frequencies of hours to days are required to measure changes in the distribution of planktonic organisms due to tidal or other circulation, phenology, or change in community structure. While the biodiversity of some structured communities like coral reefs, sea grass meadows, or mangrove forests may be expected to change more slowly, disturbance due to pollution events, severe storms, or cold or warm temperature extremes can lead to rapid changes in organism distribution, traits (e.g., bleaching), or habitat structure. High temporal resolution also increases the chance of observing targets often obscured by clouds (Mercury et al. 2012).

The proposed NASA GEOstationary Coastal and Air Pollution Events (GEO‐CAPE) mission would acquire high quality hyperspectral measurements three to four times per day of targeted tropical and subtropical coastal areas in North America, as well as opportunistically in other locations in the hemisphere of regard, but at 250–375 m spatial resolution (Salisbury et al. 2016). Furthermore, the geostationary mission would not cover high latitude areas, and more than one satellite would be required to observe other areas around the world.

Therefore, since the capability does not exist elsewhere, temporal resolution on the order of hours to days, in conjunction with the other H4 specifications, is required to adequately observe coastal zones.

## Applications and Benefits

The need for biodiversity data is expressed in international treaties, including the Convention on Biological Diversity (CBD), the U.N. Sustainable Development Goals (including SDG 6, 14, and 15; see United Nations, 2015 and Agenda, 2015), and the Ramsar Convention (MEA 2005a,2005b, WOA 2016). Similar treaties address the conservation of major freshwater bodies, such as the Laurentian Great Lakes. Of interest is using the concept of Essential Biodiversity Variables (EBVs) to monitor and assess long‐term changes in coastal ecosystems, including coastal water quality, coastal zone algal and bacterial blooms, wetlands biodiversity, benthic communities, and fishery potential. The need for global monitoring of marine biodiversity has been recognized by the Group on Earth Observations (GEO) and the Intergovernmental Oceanographic Commission (IOC; FOO 2012). GEO and the IOC have agreed to implement a Marine Biodiversity Observation Network (MBON; Duffy et al. 2013) as an integral part of the GEO BON.

In addition to meeting the objectives of these initiatives, *H4* also addresses the needs of terrestrial and fresh water studies (Schimel et al. 2015, Jetz et al. 2016, Dekker and Pinnel 2017). As a result, combining *H4* observations with those from ocean color missions, land‐observing missions, and in situ monitoring would significantly expand the scope of coastal science.

Example *H4* applications include:

1 Coastal water quality and coastal zone blooms. *H4* addresses the fundamental requirements of coastal ecology and resource monitoring programs for evaluating EBVs that inform about the quality, diversity, and productivity of coastal aquatic habitats as a function of nutrient inputs, light, and other physical and biotic factors. Specifically, H4 will provide information on:
  a Functional phytoplankton groups (red tide, coccolithophore, large and small phytoplankton cell concentration, etc.).
  b Floating vegetation (*Sargassum*, giant kelp and other large algae, sea grasses)
2 Seascapes (dynamic, multivariate biogeographic classification; e.g., Kavanaugh et al. 2016).
3 Wetland biodiversity. *H4* provides observations of wetland areal extent, canopy characteristics, species populations assemblages, and phenology, including change in emergent vegetation and water quality due to disturbance.
4 Benthic communities. *H4* monitors EBVs that track the areal extent, composition, and health of shallow subtidal foundation species (e.g., coral reef, seagrasses, kelp) and the integrity of benthic communities, in addition to providing information on shallow water bathymetry.

## Implementing *H4* Remote Sensing

Implementing a global *H4* observation system is within reach. The technology is available to obtain the required SNR for hyperspectral data at 30‐m resolution, but a single sensor in orbit cannot provide the desired revisit time for all coastal zones and inland habitats of the world. A single, agile *H4* satellite in a 3‐d repeat orbit could accommodate observations of several hundred coastal habitats distributed around the world every day, by consistently acquiring data with both along‐track (for glint mitigation) and cross‐track targeting (Osterman et al. 2016). A constellation of nine small *H4* satellites, collecting 30‐km swaths in pushbroom mode, would cover global land and coastal zones with weekly frequency. Broadening the swath would reduce the number of sensors required. Small satellite constellations are now common for a variety of applications. For example, NASA launched an eight‐satellite Cyclone Global Navigation Satellite System (CYGNSS) to measure wind speed over the ocean to improve hurricane forecasting. The Earth imaging company Planet Labs had a fleet of over 170 miniature satellites operating by mid‐2017, collecting daily data for agricultural, urban planning, disaster response, and vessel tracking applications around the world, among many other uses.

Operational resource management efforts, and an obligation to evaluate changes occurring over decadal and longer timeframes, also would require sustaining *H4* over longer periods, similar to those provided by Landsat and other operational satellite series. The *H4* observations would complement such operational satellites.

There are several strategies to increase the SNR for observations of coastal aquatic habitats and of biologically structured habitats. While one possibility is to relax the spatial resolution requirement for coastal aquatic observations to about 60–100 m to match the scales of variability in coastal aquatic properties, this is a lower resolution than required for characterizing coastal vegetation and shallow submerged habitats such as coral reefs. Binning spectrally to 6–8 nm, per recommendation of the CEOS report (2017) also helps increase SNR. A separate strategy is to alter the platform or sensor motion to scan aquatic targets slower than land or wetland targets (e.g., Osterman et al. 2016).

Aquatic measurements may be collected within a range of viewing angles (e.g., ±45°), following a strategy that mitigates sun glint. However, observations of above‐water wetland vegetation would require fixed viewing geometries to properly interpret the sequence of measurements in a time series of observations. Such off‐nadir observations also help to minimize the contaminating effects of water reflections observed through wetland canopies and help improve biomass estimates (Turpie et al. 2015).

The *H4* concept also poses challenges with respect to data downlink, management, processing, and distribution. A global coastal *H4* mission will require increased informatics, with significantly more on‐board processing and storage capacity than is typical for current science applications. Further, some monitoring applications will require near‐real‐time access to the *H4* data. Commercial companies are actively addressing such big‐data challenges with super‐high spatial resolution (<0.5 m pixels) multispectral (typically eight bands) satellite constellations. We can learn important lessons from these initiatives.

## Conclusions

The combined open ocean, coastal, and wetland *H4* observation strategy will revolutionize applied ecological research. Even one such device flown over a period of 3–5 years would enable the first comprehensive set of measurements of biodiversity variables in hundreds of coastal habitats around the world. A global *H4* observation strategy would also provide coverage of land and fresh water habitats. This can be achieved with a constellation of multiple small and low‐cost satellite sensors similar to the NASA eight‐satellite Cyclone Global Navigation Satellite System (CYGNSS) and commercial high spatial resolution imaging strategies. *H4* would define a baseline to evaluate past observations collected with less capable sensors, and to assess long‐term changes. It would enable operational assessments and management applications that sustain coastal and terrestrial ecosystem services, including provisioning of food, clean water, and economic well‐being around the world.
